# 

**Published:** 1887-07

**Authors:** 


					﻿1887.
OLD SERIES
Vol. XXV
& 18550
NEW SERIES
Vol. IV.—No. 5l ’ '
Msrf
■EDIGJL^eW
JDUB<

JE^tw
by
W.F.WESTMOREL AND. $BSj
H.VM.M1LLER. M.DJ.L .dJR
JAMES A.GRAY. MJ3.
published By jamesp.harrison&cq<
IflP _________. fflT LANTA, GA.
SUBSCRIPTION: S2.B0kA YEAR.
				

